# Broad-spectrum antibiotics associated gut microbiome disturbance impairs T cell immunity and promotes lung cancer metastasis: a retrospective study

**DOI:** 10.1186/s12885-022-10307-x

**Published:** 2022-11-17

**Authors:** Ke Xu, Jixu Cai, Jun Xing, Xu Li, Beishou Wu, Zhuxian Zhu, Ziqiang Zhang

**Affiliations:** 1 Department of General Medicine, Tongji University School of Medicine, 389 Xincun Road, Shanghai, 200065 China; 2 Jiading Community Health Service Center of Jiading District, Shanghai, 201899 China; 3 Department of Emergency Medicine, Tongji University School of Medicine, 389 Xincun Road, Shanghai, 200065 China; 4 Department of Infectious Disease, Tongji Hospital, Tongji University School of Medicine, 389 Xincun Road, Shanghai, 200065 China; 5 Department of Nephrology, Tongji Hospital, Tongji University School of Medicine, 389 Xincun Road, Shanghai, 200065 China; 6 Department of Respiratory and Critical Care Medicine, Tongji Hospital, Tongji University School of Medicine, 389 Xincun Road, Shanghai, 200065 China

**Keywords:** Gut microbiota, Lung cancer, Metastasis, T cell immunity, Broad-spectrum antibiotics (ATB)

## Abstract

**Background:**

Gut microbiome has been linked to a regulatory role in cancer progression. However, whether broad-spectrum antibiotics (ATB) associated gut microbiome dysbiosis contributes to an impaired T cell immune function, and ultimately promotes lung cancer metastasis is not well known.

**Methods:**

In this study, a retrospective analysis was performed in a cohort of 263 patients initially diagnosed with non-small cell lung cancer (NSCLC) patients, including the ATB group (patients with broad-spectrum antibiotics treatment) (*n* = 124), and non-ATB group (*n* = 139) as control. ATB patients were prescribed ATB for over 5 days within 30 days prior to the collection of blood and fecal specimens and followed surgical treatment or first-line therapy. T cell immune function and metastasis-free survival (MFS) were evaluated between the two groups. Gut microbiota was evaluated by 16S rDNA sequencing. The predictive value of T cell immunity for MFS was evaluated by ROC analysis and Cox regression analysis.

**Results:**

Our results suggest that broad-spectrum antibiotics (ATB) impair T cell immune function in patients with either early-stage or advanced NSCLC, which likely contribute to the promotion of lung cancer metastasis. Results of the survival analysis show that metastasis-free survival (MFS) is significantly shorter in the ATB patients than that in the non-ATB patients with stage III NSCLC. The 16S rDNA sequencing shows that ATB administration contributes to a significant dysbiosis of the composition and diversity of gut microbiota. Moreover, ROC analysis results of CD4 (AUC 0.642, *p* = 0.011), CD8 (AUC was 0.729, *p* < 0.001), CD16 + 56 + (AUC 0.643, *p* = 0.003), and the combination of CD4, CD8 and CD16 + 56+ (AUC 0.810, *p* < 0.001), or Cox regression analysis results of CD4 (HR 0.206, *p* < 0.001), CD8 (HR 0.555, *p* = 0.009), which is likely regulated by ATB administration, have significantly predictive values for MFS.

**Conclusion:**

These results provide evidence of gut microbiome disturbance due to ATB administration is involved in the regulation of T cell immunity, and their predictive value for the tumor metastasis in lung cancer patients. Thus, gut microbiota may serve as a therapeutic target for lung cancer. Consequently, caution should be exercised before the long-term administration of broad-spectrum antibiotics in cancer patients.

## Background

Human microecosystem is associated with the regulation of immune system. Studies have explored how lung microbiota influences cancer outcome [1–5], Abnormal gut microbiome composition may attribute to cancer progression [6–11]. For example, gut bacteria are involved in the regulation of tumor treatment responses [12–15]. Bifidobacterium administration contributes to the enhancement of anti-cancer immunity, and blocks the melanoma growth [13]. Moreover, a recent study reported that anti-cancer role of gut microbiota, such as the Clostridiales members, are associated with the activation of tumoral CD8+ T cells [16]. These results, either in preclinical murine models or human studies, have highlighted the importance of gut microbiota in the regulation of anti-cancer therapeutics, and thus help to develop better therapeutic strategies by modulating gut microbiota.

Cancer patients receive broad-spectrum antibiotics (ATB) generally for common indications (such as pneumonia or urinary tract infection) [17, 18], or to exclude infectious diseases before the final diagnosis of cancer. However, studies have suggested that ATB represented a predictor of resistance to chemotherapy [6]. Antibiotics also inhibit the benefits of immunotherapy in patients with advanced cancer [14, 19], In addition, ATB can alter the composition of gut microbiota [20–22]. Thus, maintaining a healthy gut microbiome may help patients combat cancer.

Accumulating evidence has indicated that gut microbiota is associated with cancer development. Probiotics can remodel the tumor microenvironment, including reducing inflammatory T helper cells and the differentiation of regulatory T cells (Treg cells) [23], or promoting the maturation of dendritic cells [24], and subsequently enhancing the response of antigen-specific cytotoxic T lymphocyte (CTL) and cancer immune surveillance. For example, Lactobacillus bacteria can improve the treatment response of cisplatin in murine cancer model [25]. Thus, it is possible to improve the therapeutic response by modulating the gut microbiome [26–28].

As a new hallmark of cancer, microbiota has caught a great attention in recent years [13]. In this regard, gut microbiota may have been considered as a potential biomarker for cancer diagnosis, treatment, and prognosis. However, it is largely unknown whether gut microbiota disturbance due to ATB contributes to an impaired T cell immune function, ultimately promoting lung cancer metastasis. In this study, we retrospectively analyzed the clinical data in a cohort of NSCLC patients with or without receiving ATB, and evaluated the effect of ATB on gut microbiota. We also performed T cell immune function, and ROC analysis and Cox regression analysis for the prediction of MFS.

## Patients and methods

### Patients and clinical data collection

**Fig. 1:**
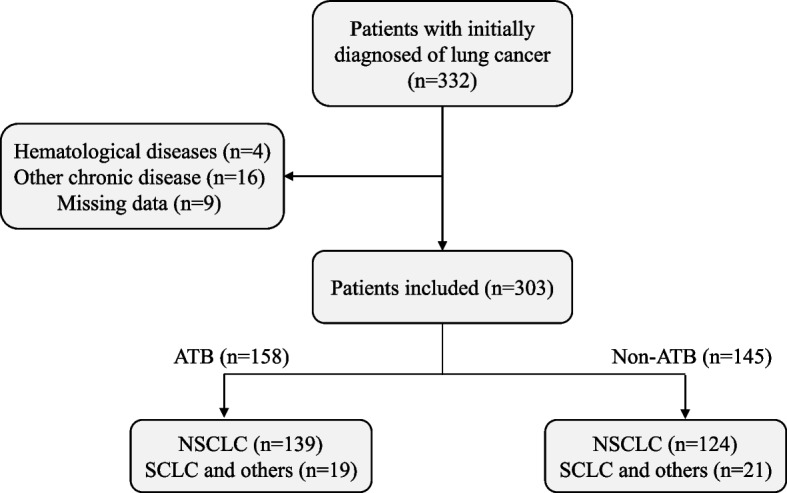
Flow diagram of this study

Data of patients diagnosed with lung cancer from Tongji Hospital of Tongji University in Shanghai China, from January 2016 to November 2021, were collected in this retrospective study. Individuals with any tumors other than lung cancer were excluded from this study. All patients were diagnosed by cytological and/or histological examination according to the WHO classification. The laboratory data of patients were collected. Total 332 lung cancer patients were included in this study, patients with Hematological diseases or with missing data were excluded. Finally, a cohort of 303 patients with initially diagnosed of lung cancer was included in the study, including 263 NSCLC patients (Fig. 1). Demographics and clinical characteristics, including age, gender, pathology, and clinical stage, were collected. Patient characteristics, such as the smoking status of patients included in this study were obtained. The smoking history of patients included in this study was obtained via in-patient history recording or interview using a questionnaire. All procedures performed in this study involving human participants were following the Declaration of Helsinki (as revised in 2013).

Among the cohort of 303 patients, 145 patients, including 124 NSCLC patients, were prescribed an intravenous infusion of broad-spectrum antibiotics (ATB). ATB administration was performed because of the diagnostic treatment to exclude infectious diseases in the suspected infection patients, or the infection patients due to common indications (combined with pneumonitis). ATB group of patients received ATB therapy for over 5 days within 30 days prior to the collection of blood and fecal specimens on admission and followed surgical treatment or first-line therapy. The other 158 lung cancer patients, including 139 NSCLC patients who did not receive antibiotics treatment as control. The blood and fecal specimens of all the patients were collected for the evaluation of T immune cells and gut microbiome prior to the surgical treatment or first-line therapy. Kaplan-Meier estimates for metastasis-free survival (MFS) of patients with stage III lung cancer were performed. All the stage III NSCLC patients were initially diagnosed of lung cancer. The MFS evaluation of stage III patients was during the period from the initial treatment until the development of metastasis. Moreover, fecal specimens of 22 out of these 303 patients were collected for 16S rDNA sequencing.

Clinical data of patients were collected, including age, gender, ECOG value, tumor stage, pathological type of tumor, and smoking status et al. Data of laboratory tests including white blood cell count, neutrophil count, lymphocyte count, C-reactive protein, platelet count, D-dimer, and T cell series et al. were collected. This study was approved by the Ethics Committee of Tongji Hospital, Tongji University (No. K-KYSB-2020-189). Informed consent was signed by the participants or their authorized family members.

### 16S rDNA sequencing

DNA extraction and PCR amplification as described in our previous study [29]: Bacterial DNA was extracted using the E.Z.N.A.® Soil DNA Kit (Omega Bio-Tek, Norcross, U.S.) from mouse feces specimens. We amplified the V4-V5 region of the bacteria 16S ribosomal RNA gene by PCR, and using primers 515F 5′-barcode- GTGCCAGCMGCCGCGG)-3′ and 907R 5′-CCGTCAATTCMTTTRAGTTT-3′. The PCR amplification conditions were:95 °C for 2 min, followed by 25 cycles at 95 °C for 30s, 55 °C for 30s, and 72 °C for 30s, and a final extension at 72 °C for 5 min. PCR reactions were performed as described previously [29]. Amplicons were extracted from 2% agarose gels and purified according to the manufacturer’s instructions. Library Construction and Sequencing: The purified PCR products were quantified by Qubit®3.0 (Life Invitrogen). We used the pooled DNA product to construct the Illumina pair-end library by following the Illumina’s genomic DNA library preparation procedure. Then this constructed amplicon library was paired-end sequenced (2 × 250) by an Illumina HiSeq platform (Shanghai BIOZERON Co., Ltd) as described previously [29], according to the standard protocols.

### Clinical outcomes

The patient’s performance status (PS score) was assessed by the Eastern Cooperative Oncology Group (ECOG). The scoring criteria divide patients’ activity status into 6 levels ranging from 0 to 5: asymptomatic (PS 0); symptomatic but completely ambulatory (PS 1); symptomatic, and < 50% in bed during the day (PS 2); symptomatic and > 50% in bed (PS 3); bedbound (PS 4); and death (PS 5).

### Statistical methods

According to the same type of study [30], the test efficiency is 0.8, the sample size included in this study meets the statistical requirements. Descriptive analyses were performed with either means ± standard deviation (continuous variables) to describe the patient’s characteristics. Continuous variables were compared by rank-sum test and T-test. The receiver operating characteristic (ROC) curve was calculated from the logistic regression model. The area under the curve (AUC) was used to evaluate the strength of prediction. Using the ROC curve to analyze the levels of CD4+ T cells, CD8+ T cells, CD16 + 56+ T cells, and D-Dimer to predict the best truncation value of MFS in patients with stage III NSCLC [determined by Youden index, Yordan index = sensitivity + specificity-1, the best truncation value is taken at the maximum of Yoden index]. All statistical analyses were performed using SPSS (version 23.0). A two-sided *p*-value < 0.05 was considered as statistically significant.

## Results

### Baseline characteristics of lung cancer patients

The clinical characteristics of 303 lung cancer patients enrolled in this study were presented in Table 1. Compared with 158 lung cancer patients without broad-spectrum antibiotics (ATB) treatment, 145 lung cancer patients were prescribed ATB. Patients with NSCLC were treated by the standard lung cancer therapy scheme.

**Table 1:** Basline clinical data of 303 patients with lung cancer

Demographics/anthropometric	Non-ATB(*n* = 158)	ATB(*n* = 145)	***P*** value
Asian	158	145	
Age (yr, mean ± SD)	69.35 ± 10.14	71.31 ± 10.71	0.104
Sex			0.479
Male (No.)	119 (75.32%)	104 (71.72%)	
Female	39 (24.68%)	41 (28.28%)	
ECOG			0.090
0–2	144 (91.14%)	123 (84.83%)	
> 2	14 (8.86%)	22 (15.17%)	
Tumor stage (%)			0.788
I	20 (12.66%)	18 (12.41%)	
II	15 (9.49%)	16 (11.03%)	
III	112 (70.89%)	55 (37.93%)	
IV	11 (6.96%)	56 (38.62%)	
Tumor type (%)			0.528
ADC	94 (59.49%)	86 (59.31%)	
SCC	45 (28.48%)	38 (26.21%)	
SCLC	12 (7.59%)	10 (6.90%)	
others	7 (4.43%)	11 (7.59%)	
Smoking status			0.764
Never smoker	79 (50%)	70 (48.28%)	
ever smoker	79 (50%)	75 (51.72%)	

*ADC* Adenocarcinoma, *SCC* Squamous cell carcinoma, *SCLC* small cell lung cancer

In the ATB patients, the mean age was 71.31 years, and 71.72% of the patients were male, 124 out of 145 ATB patients were NSCLC, and there was 23.44% for stage I-II and 76.55% for stage III-IV. In the non-ATB patients, the mean age was 69.35 years, and 75.32% of the patients were male, 145 out of 158 non-ATB patients were NSCLC, and there was 22.51% for stage I-II and 77.85% for stage III-IV. (Table 1).

### Antibiotics administration associated with enhanced cancer metastasis

To determine the impact of ATB on patients with advanced NSCLC, we performed the analysis for a cohort of 143 patients with stage III NSCLC out of the above 303 lung cancer patients. Among them, 47 patients have prescribed an intravenous infusion of ATB (ATB group, *n* = 47), and the other 96 patients did not receive antibiotics treatment (non-ATB group, *n* = 96). The demographic and clinical characteristics of 143 lung cancer patients with stage III NSCLC are present in Table 2. After the initial diagnosis of lung cancer, the patients received standard anti-cancer therapy.

**Table 2:** Baseline clinical data of 143 patients with in patients with stage III NSCLC

Demographics/anthropometric	Non-ATB(*n* = 96)	ATB(*n* = 47)	***P*** value
Asian	96	47	
Age (yr, mean ± SD)	70.96 ± 10.27	74.49 ± 11.41	0.062
Sex			0.548
Male (No.)	70 (72.92%)	32 (68.09%)	
Female	26 (27.08%)	15 (31.91%)	
ECOG			0.709
0–2	86 (89.58%)	37 (78.72%)	
> 2	10 (10.42%)	10 (21.28%)	
Tumor type (%)			0.158
ADC	56 (58.33%)	25 (53.19%)	
SCC	40 (41.67%)	22 (46.81%)	
Therapeutic typology			0.081
Surgery	8 (8.33%)	6 (12.77%)	
Chemotherapy	55 (57.3%)	34 (72.3%)	
Targeted therapy	34 (35.4%)	6 (12.8%)	
Immunotherapy	9 (9.4%)	3 (6.4%)	
Radiotherapy	5 (5.2%)	6 (12.8%)	
Treatment line			0.721
First line	78 (81.25%)	37 (78.7%)	
Subsequent lines	18 (18.75%)	10 (21.3%)	
Smoking status			0.916
Never smoker	54 (56.25%)	26 (55.32%)	
ever smoker	42 (43.75%)	21 (44.68%)	

**Fig. 2:**
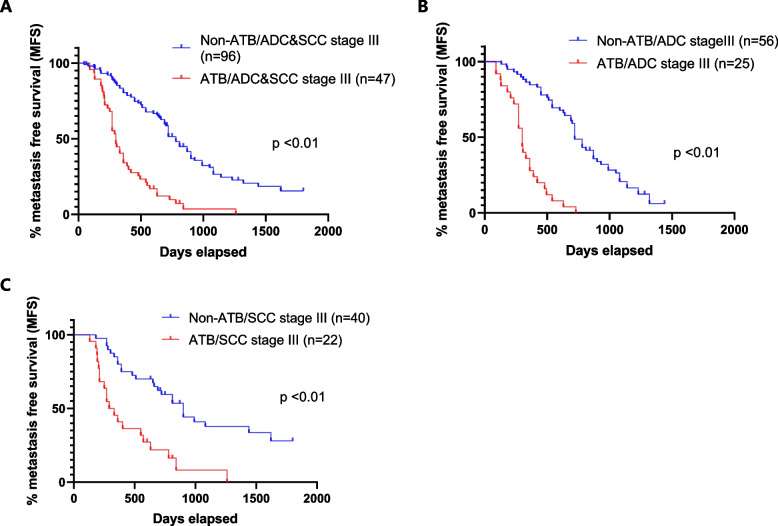
Kaplan-Meier estimates for the metastasis-free survival (MFS) in stage III NSCLC patients treated with or without ATB administration, according to the pathological types. A: adenocarcinoma (ADC) & squamous carcinoma (SCC) (**A**), ADC (**B**), SCC (**C**)

In this study, it was evident that ATB promoted lung cancer metastasis. Metastasis-free survival (MFS) was significantly shorter in the ATB group than that in the non-ATB group. (Fig. 2A). The influences of ATB on metastasis were further evaluated according to the pathological types (adenocarcinoma or squamous carcinoma), and the results showed that ATB administration significantly promotes tumor metastasis in either adenocarcinoma or squamous cell carcinoma of lung cancer (Fig. 2B-C).

### Evaluation of gut microbiota by 16S rDNA sequencing

**Fig. 3:**
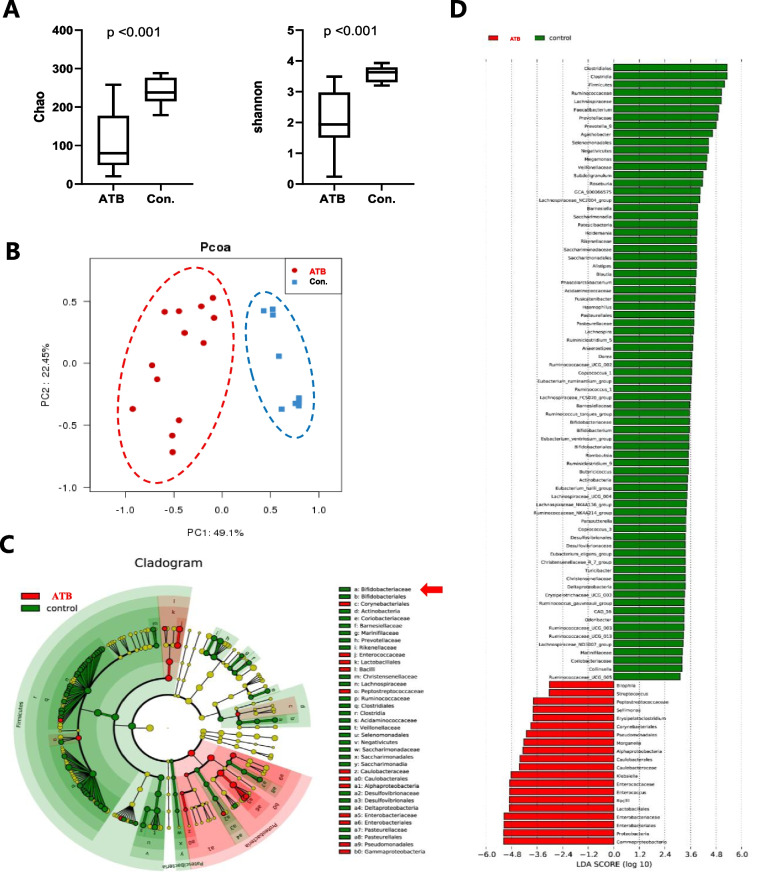
The composition and diversity of the gut microbiota of fecal specimens from stage III lung cancer patients treated with or without ATB. Alpha diversity (Chao, Shannon index) between the ATB and control (**A**). Principal coordinate analysis (PCoA) using weighted-UniFrad of beta diversity (**B**). Taxonomic Cladogram from LEfSe, depicting taxonomic association between microbiome communities from the two groups (**C**). LDA score computed from features differentially abundant between the two groups (**D**)

To evaluate the taxonomic composition and microbial diversity of gut microbiome between the ATB and non-ATB lung cancer patients, which might influence tumor metastasis, alpha and beta diversity were analyzed. The results of alpha diversity (Chao and Shannon index), which reflect the species richness and diversity, were significantly higher in the non-ATB than that in the ATB group (Fig. 3A). To compare the composition of the microbial community between the two groups, we used beta diversity to generate the weighted UniFrac principal coordinates analysis (PCoA) and showed the clustering between non-ATB and the ATB patients, as shown in Fig. 3B.

To identify the specific microbial communities associated with ATB treatment, we analyzed the composition of the gut microbiota by using LEfSe analysis. A total of 37 discriminative taxa at all taxonomic levels from phylum to genus were identified (LDA > 3, *p* < 0.05). At the phylum level, the abundance of Bifidobacteriaceae, Actinobacteria, and Coriobacteriaceae were enriched in the non-ATB patients, whereas.

**Fig. 4:**
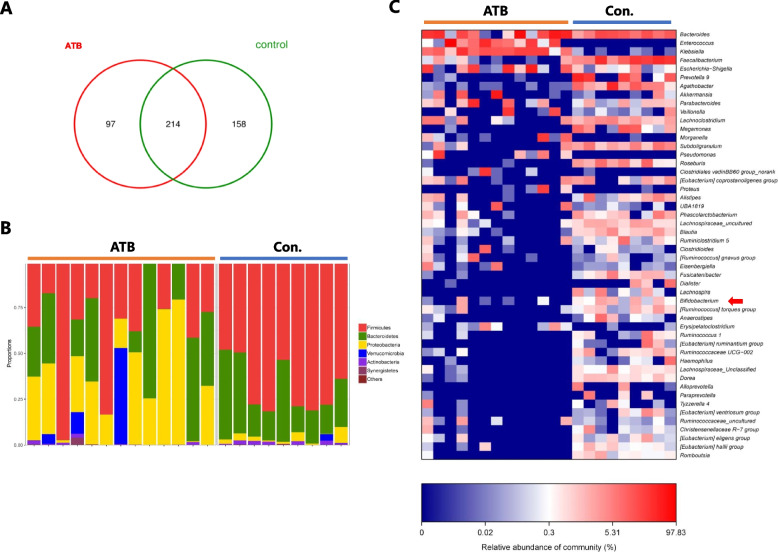
The composition of the gut microbiota of fecal specimens from stage III lung cancer patients. The Venn diagram illustrates the overlapped OTUs (**A**). Bar plots of the class taxonomic levels (**B**). Heatmap of selected most differentially abundant features at the genus level (**C**), Arrowed lines highlighting the taxa enriched in the fecal samples of the control group

Gammaproteobacteria, Enterobacteriaceae, and Corynebacteriales was enriched in the ATB group (Fig. 3C-D). Moreover, as shown in the Venn diagram, 311 and 372 OTUs were detected in the ATB and non-ATB (control) groups, respectively, with 214 OTUs concurrent in the two groups (Fig. 4A). Bar plots of the class taxonomic levels in the two groups were shown in Fig. 4B. At the genus level, the abundance of Bifidobacterium, Faecalibacterium, and Agathobacter were significantly decreased in the ATB group, compared with the non-ATB patients (Fig. 4C). The 16S rDNA sequencing data have been deposited to the NCBI Sequence Read Archive (SRA) database (Accession Number: SRP226777).

### Effects of broad-spectrum antibiotics on T cell immune function

The association of T cell subsets and the use of antibiotics is shown in Table 3. We firstly evaluated all the 303 lung cancer patients in this study, the results showed that CD3, CD4, CD8, and CD16 + 56+ T cells were significantly decreased in the ATB group (*n* = 145) than that in the non-ATB group (*n* = 158) (*p* < 0.01). In addition, Lymphocyte ratio (L%), NLR, and D-dimer were also significantly altered between the two groups. However, there was no significant difference in CD19, C3, IgG, IgA, IgM, and C4 between the two groups.

**Table 3:** Analysis of immune function and laboratory indexes in 303 patients with lung cancer

Characteristic	Non-ATB (*n* = 158)	ATB (*n* = 145)	***P*** value
CD3+ (%)	69.60 (63.96，75.70)	65.50 (55.25，71.00)	< 0.001
CD4+ (%)	43.20 ± 10.49	34.81 ± 10.98	< 0.001
CD8+ (%)	23.30 (17.02，28.30)	20.30 (15.34，24.25)	< 0.001
CD4/CD8	1.90 (1.37，2.57)	1.70 (1.20，2.25)	0.146
CD16 + 56+ (%)	14.80 (10.10，21.75)	18.00 (12.60，25.05)	0.002
CD19+ (%)	9.36 (6.50，13.38)	9.50 (6.00，15.05)	0.709
C3 (g/L)	1.06 (0.87，1.21)	1.07 (0.90，1.22)	0.523
IgG (g/L)	12.25 (10.22，14.58)	11.95 (9.73，15.08)	0.837
IgA (g/L)	2.54 (1.84，3.17)	2.67 (1.77，3.71)	0.349
IgM (g/L)	0.86 (0.63，1.27)	0.84 (0.55，1.09)	0.110
C4 (g/L)	0.26 (0.21，0.33)	0.26 (0.19，0.31)	0.376
WBC(*10^9/L)	8.64 (6.11，11.37)	9.27 (6.78，13.51)	0.071
L%	16.70 (11.00，23.40)	12.90 (8.20，20.20)	0.003
NLR	4.42 (2.77，6.95)	5.68 (3.53，10.40)	0.003
PLT(*10^9/L)	239.00 (183.50，291.50)	221.00 (178.00，311.00)	0.853
CRP (mg/L)	20.16 (4.05，68.36)	27.73 (7.28，94.99)	0.045
PCT (ng/L)	1.64 (0.11，4.37)	2.13 (0.82，4.85)	0.076
D-Dimer (mg/L)	1.03 (0.49，2.67)	2.46 (0.93，5.21)	< 0.001

*WBC* white blood cell, *L%* Percentage of lymphocytes, *NLR* Neutrophil-lymphocyte ratio, *PLT* platelet, *CRP* C-reactive protein, *PCT* Procalcitonin, *C3* Complement C3, *IgG* Immunoglobulin G, *IgA* Immunoglobulin A, *IgM* Immunoglobulin M, *C4* Complement C4

Next, according to the 263 NSCLC patients out of the above 303 patients, including ATB patients (*n* = 124) and non-ATB patients (*n* = 139). Our result showed that CD4, CD8, and CD16 + 56+ T cells, and L% were significantly decreased in the ATB group than that in the non-ATB group (*p* < 0.05) (Table 4). In the early stage of NSCLC patients, CD4, and CD8 T cells were significantly lower in the ATB group (*n* = 29) than that in the non-ATB group (*n* = 35) (*p* < 0.05) (Table 5). In the advanced stage of NSCLC patients, CD3, CD4, CD8, and CD16 + 56+ T cells were significantly decreased, while D-Dimer was significantly increased in the ATB group (*n* = 95) than that in the non-ATB group (*n* = 104) (*p* < 0.05) (Table 6). In ADC patients, CD3, CD4, CD8, and CD16 + 56+ T cells were significantly decreased, while D-Dimer was significantly elevated in the ATB group(*n* = 86) than in the non-ATB group (*n* = 94) (*p* < 0.05) (Table 7). In SCC patients, CD3, CD4, CD8, and CD16 + 56+ T cells were significantly decreased, while D-Dimer was significantly elevated in the ATB group (*n* = 38) than in the non-ATB group (*n* = 45) (*p* < 0.05) (Table 8).

**Table 4:** Analysis of T cell subsets, IgM, L%, NLR, CRP and D-Dimer in 263 NSCLC patients

Characteristic	Non-ATB(*n* = 139)	ATB(*n* = 124)	***P*** value
CD3+ (%)	69.65 (64.68，63.68)	63.79 (54.69，70.33)	< 0.001
CD4+ (%)	42.93 ± 10.54	34.53 ± 11.01	< 0.001
CD8+ (%)	23.30 (17.10，28.30)	20.30 (15.58，23.63)	< 0.001
CD4/CD8	1.88 (1.34，2.52)	1.67 (1.21，2.30)	0.261
CD16 + 56+ (%)	14.84 (10.19，22.23)	18.88 (12.73，26.10)	0.001
CD19+ (%)	8.90 (6.18，13.22)	9.12 (6.03，14.43)	0.662
IgM (g/L)	0.86 (0.59，1.26)	0.84 (0.56，1.08)	0.124
L%	16.15 (11.20，23.18)	13.40 (8.90，20.20)	0.016
NLR	4.52 (2.89，6.85)	5.64 (3.50，9.29)	0.013
CRP (mg/L)	19.31 (4.23，60.76)	24.60 (6.89，92.60)	0.077
D-Dimer (mg/L)	1.09 (0.51，2.80)	2.56 (0.94，5.38)	< 0.001

*L%* Percentage of lymphocytes, *NLR* Neutrophil-lymphocyte ratio, *CRP* C-reactive protein, *IgM* Immunoglobulin M

**Table 5:** Analysis of T cell subsets, IgM, L%, NLR, CRP and D-Dimer in early-stage (stage I & II) NSCLC patients

Tumor stage	Non-ATB(*n* = 35)	ATB(*n* = 29)	***P*** value
I	20	16	36
II	15	13	28
CD3+ (%)	69.20 (62.50，74.53)	67.10 (56.90，72.25)	0.133
CD4+ (%)	40.81 ± 11.11	28.03 ± 10.05	< 0.001
CD8+ (%)	28.46 ± 8.96	21.06 ± 7.59	0.001
CD4/CD8	1.57 ± 0.60	1.50 ± 0.78	0.712
CD16 + 56+ (%)	14.84 ± 8.09	18.23 ± 9.55	0.129
CD19+ (%)	8.70 (6.71，13.40)	10.45 (5.42，17.22)	0.936
IgM (g/L)	1.03 ± 0.40	0.95 ± 0.33	0.467
L%	18.10 (10.80，28.20)	16.00 (10.80，20.20)	0.328
NLR	3.52 (2.09，6.85)	4.28 (3.52，7.67)	0.254
CRP (mg/L)	8.03 (2.73，41.48)	14.56 (2.26，134.93)	0.307
D-Dimer (mg/L)	0.85 (0.31，2.80)	1.60 (0.74，4.55)	0.131

*L%* Percentage of lymphocytes, *NLR* Neutrophil-lymphocyte ratio, *CRP* C-reactive protein, *IgM* Immunoglobulin M

**Table 6:** Analysis of T cell subsets, IgM, L%, NLR, CRP and D-Dimer in advanced stage (stage III & IV) NSCLC patients

Tumor stage	Non-ATB (*n* = 104)	ATB(*n* = 95)	***P*** value
III	96	47	
IV	8	48	
CD3+ (%)	69.90(64.60，76.10)	63.20 (53.50，69.50)	< 0.001
CD4+ (%)	43.65 ± 10.30	36.52 ± 10.56	< 0.001
CD8+ (%)	21.71 (16.00，27.80)	20.10 (15.27，23.80)	0.031
CD4/CD8	1.99 (1.39，3.01)	1.79 (1.35，2.38)	0.222
CD16 + 56+ (%)	14.87 (10.30，22.70)	19.10 (13.30，29.40)	0.002
CD19+ (%)	9.10 (5.90，13.15)	9.00 (6.21，12.70)	0.596
IgM (g/L)	0.82 (0.58，1.29)	0.83 (0.55，1.03)	0.162
L%	16.10 (11.20，22.70)	12.40 (8.30，20.20)	0.027
NLR	4.65 (2.93，6.85)	5.88 (3.51，10.16)	0.028
CRP (mg/L)	22.82 (4.63，66.39)	31.59 (7.47，79.03)	0.177
D-Dimer (mg/L)	1.15 (0.59，2.77)	3.18 (1.05，5.42)	< 0.001

*L%* Percentage of lymphocytes, *NLR* Neutrophil-lymphocyte ratio, *CRP* C-reactive protein, *IgM* Immunoglobulin M

**Table 7:** Analysis of T cell subsets, IgM, L%, NLR, CRP and D-Dimer in adenocarcinoma (ADC) patients

Characteristic (ADC)	Non-ATB (*n* = 94)	ATB(*n* = 86)	***P*** value
CD3+ (%)	68.80 (63.24, 76.35)	64.28 (53.85, 70.93)	< 0.001
CD4+ (%)	42.99 ± 10.41	35.00 ± 11.33	< 0.001
CD8+ (%)	23.90 (17.60，28.20)	19.90 (15.00，24.69)	0.001
CD4/CD8	1.93 (1.41，2.46)	1.74 (1.26，2.28)	0.549
CD16 + 56+ (%)	15.03 (10.35，22.25)	16.90 (12.60，24.69)	0.029
CD19+ (%)	9.36 (6.20，13.34)	9.50 (5.88，15.48)	0.740
IgM (g/L)	0.81 (0.57，1.20)	0.86 (0.57，1.18)	0.821
L%	16.50 (10.70，23.35)	13.35 (8.68，20.20)	0.106
NLR	4.44 (2.79，6.95)	5.57 (3.49，9.62)	0.096
CRP (mg/L)	19.06 (2.66，41.63)	13.79 (3.33，61.89)	0.726
D-Dimer (mg/L)	1.26 (0.63，4.20)	2.92 (1.11，6.01)	0.002

*L%* Percentage of lymphocytes, *NLR* Neutrophil-lymphocyte ratio, *CRP* C-reactive protein *IgM* Immunoglobulin M

**Table 8:** Analysis of T cell subsets, IgM, L%, NLR, CRP and D-Dimer in squamous cell carcinoma (SCC) patients

Characteristic (SCC)	Non-ATB (*n* = 45)	ATB(*n* = 38)	***P*** value
CD3+ (%)	69.78 ± 9.10	62.61 ± 9.74	0.001
CD4+ (%)	42.81 ± 10.93	33.47 ± 10.33	< 0.001
CD8+ (%)	25.03 ± 10.51	20.90 ± 5.02	0.030
CD4/CD8	1.82 (1.18，2.81)	1.62 (1.18，2.32)	0.315
CD16 + 56+ (%)	15.76 ± 8.10	22.25 ± 10.21	0.002
CD19+ (%)	8.10 (3.98，12.25)	8.30 (6.16，11.49)	0.742
IgM (g/L)	0.91 (0.69，1.45)	0.77 (0.55，1.00)	0.009
L%	18.18 ± 8.95	14.29 ± 6.97	0.032
NLR	4.33 (2.72，6.65)	5.67 (3.59，8.92)	0.040
CRP (mg/L)	23.26 (5.40，70.07)	76.97 (26.06，117.10)	0.001
D-Dimer (mg/L)	0.79 (0.41，1.42)	2.37 (0.64，5.21)	0.003

*L%* Percentage of lymphocytes, *NLR* Neutrophil-lymphocyte ratio, *CRP* C-reactive protein, *IgM* Immunoglobulin M

### Predictive value of T cell immunity for metastasis-free survival (MFS) in the NSCLC patients

**Fig. 5:**
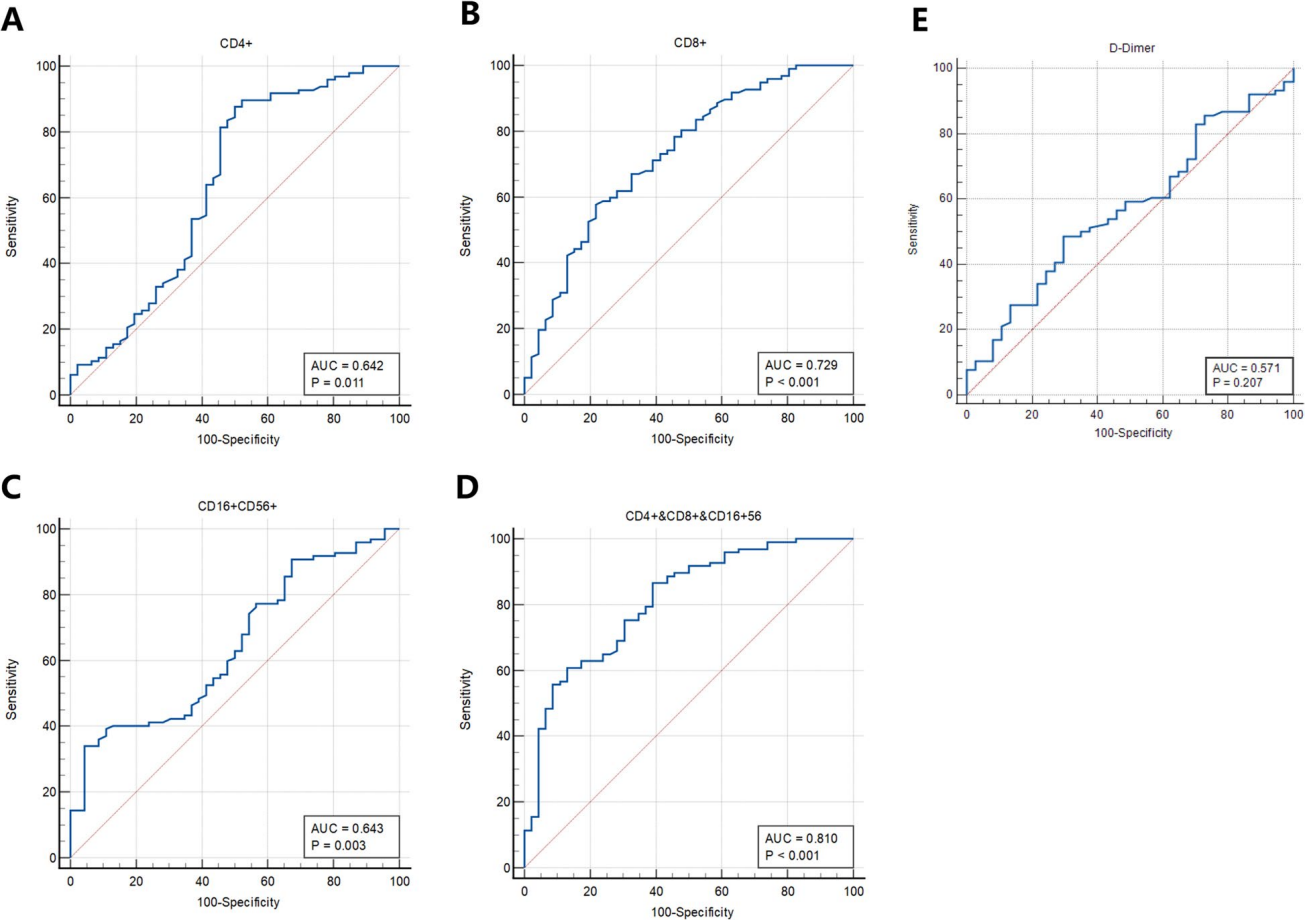
The predictive value of CD4 (**A**), CD8 (**B**), CD16/56 (**C**), combination of CD4, CD8, and CD16/56 (**D**), and D-Dimer (**E**) for Metastasis-free survival (MFS) by ROC analysis

ROC analysis was used to calculate the area under the curve (AUC) of CD8 T cell, CD4 T cell, CD16 + 56+, D-Dimer, and MFS in 143 NSCLC patients with stage III lung cancer (ATB, *n* = 47; non-ATB, *n* = 96). Our results showed that the AUC of CD4 T cell was 0.642 (*p* < 0.001) (Fig. 5A), the AUC of CD8 T cells was 0.729 (*p* < 0.001) (Fig. 5B), the AUC of CD16 + 56+ T cells was 0.643 (*p* < 0.05), (Fig. 5C), the AUC of combined CD4, CD8 and CD16 + 56+ T cells was 0.810 (*p* < 0.001), (Fig. 5D). While the AUC of D-dimer did not demonstrate significant predictive values for MFS (*p* = 0.201) (Fig. 5E). Thus, the AUC of CD4, CD8, and the combined group demonstrated significantly predictive values for MFS, and the prediction value of the combination of CD4, CD8, and CD16 + 56+ T cells is particularly significant. (Fig. 5 and Table 9).

**Table 9:** ROC analysis: the predictive role of ATB regulated T cell immunity for metastasis-free survival (MFS) in stage III NSCLC patients

Characteristic	AUC	*P* value	95%CI
CD4+	0.642	0.011	0.557 ~ 0.720
CD8+	0.729	< 0.001	0.649 ~ 0.800
CD16 + 56+	0.643	0.003	0.559 ~ 0.721
CD4+ &CD8 + &CD16 + 56	0.810	< 0.001	0.736 ~ 0.870
D-Dimer	0.571	0.201	0.475 ~ 0.664

### Univariate and multivariate cox regression analysis of the risk factors on MFS in patients with stage III NSCLC

In this study, the Univariate analysis showed that age, ATB administration, CD4+, CD8+ and CD16 + 56+ T cell levels, but not sex, smoking status, tumor pathological type, or D-Dimer, are associated with MFS in patients with stage III NSCLC (Table 10). The risk factors (*p* < 0.05) in the univariate analysis were included in the subsequently multivariate Cox regression analysis. The Cox regression analysis showed that only CD4 T cells or CD8 T cells is significantly associated with MFS in the patients with stage III NSCLC (Table 11). These results suggest that CD4+ or CD8+, but not ATB administration itself, are the independent risk factors for MFS in patients with stage III NSCLC. Thus, the disturbance of gut microbiome due to ATB administration, but not antibiotic application itself, may be directly involved in the regulation of T cell immunity, and ultimately influence the metastasis-free survival. However, further studies are needed.

**Table 10:** Univariate Analysis of the risk factors associated with MFS in patients with stage III NSCLC

Factor	n,(%)	*Χ*^*2*^	***P***
Sex		0.103	0.748
Male	102		
Female	41		
Age (year)		5.438	0.020
≤ 65	47		
>65	96		
Smoking status		0.208	0.720
Ever smoker	73		
Never smoker	80		
Tumor type		0.380	0.538
SCC	60		
ADC	83		
Use of ATB		14.181	< 0.001
ATB	96		
Non-ATB	47		
CD4+ (%)		21.643	< 0.001
>32.10	109		
≤ 32.10	34		
CD8+ (%)		15.195	< 0.001
>22.10	68		
≤ 22.10	75		
CD16 + 56+ (%)		14.124	< 0.001
>10.88	109		
≤ 10.88	34		
D-Dimer (mg/L)		2.617	0.106
>1.04	73		
≤ 1.04	70		
Treatment modalities		2.087	0.539
Surgery	14		
Chemotherapy	89		
Targeted therapy	40		
Immunotherapy	12		
Radiotherapy	11

**Table 11:** Cox regression analysis of multiple risk factors associated with MFS in patients with stage III NSCLC

Factor	B	SE	Wald	***P***	HR	95%CI
Age	0.153	0.234	0.430	0.512	1.166	0.737 ~ 1.843
Use of ATB	0.180	0.255	0.499	0.480	1.198	0.726 ~ 1.975
CD4+ (%)	−1.582	0.357	19.647	< 0.001	0.206	0.102 ~ 0.414
CD8+ (%)	−0.589	0.226	6.447	0.009	0.555	0.356 ~ 0.865
CD15 + 56+ (%)	−0.167	0.223	0.560	0.454	0.846	0.546 ~ 1.311

### Effect of treatment line and typology on T cell immunity in patients with stage III NSCLC

In this study, we further evaluated CD4 T cells and CD8 T cells in patients who received first-line therapy or subsequent lines therapy, between the non-ATB and ATB groups. CD4 (*p* = 0.004) and CD8 T cells (*p* = 0.003) in patients who received first-line therapy, and CD4 (*p* = 0.035) and CD8 T cells (*p* = 0.181) in patients who received subsequent lines therapy were found between the non-ATB and ATB groups. We also evaluated CD4 T cells and CD8 T cells in patients who only received chemotherapy or patients who received other treatments (including Immunotherapy, targeted therapy or radiotherapy, which were used individually or in combination), between the non-ATB and ATB groups. CD4 (*p* = 0.002) and CD8 T cells (*p* = 0.171) in patients who only received chemotherapy therapy, and CD4 (*p* = 0.033) and CD8 T cells (*p* = 0.376) in patients who received other treatments were found between the non-ATB and ATB groups. (Table 12).

**Table 12:** Effect of treatment line or therapeutic regimen on T cell immunity in patients with stage III NSCLC

Treatment	Item	Non-ATB (*n* = 78)	n	ATB (*n* = 37)	n	*P* value
First line	CD4+	42.83 ± 9.36	78	37.08 ± 10.28	37	0.004
	CD8+	23.15 ± 7.42	78	19.10 ± 5.14	37	0.003
Subsequent lines	CD4+	40.62 ± 14.70	18	34.49 ± 9.19	10	0.035
	CD8+	22.76 ± 9.65	18	23.29 ± 6.17	10	0.181
Chemotherapy	CD4+	44.10 ± 9.81	55	36.95 ± 11.07	34	0.002
	CD8+	22.68 ± 7.55	55	19.46 ± 5.36	34	0.033
Other treatment	CD4+	40.16 ± 11.11	41	35.45 ± 8.96	13	0.171
	CD8+	23.60 ± 8.27	41	21.38 ± 6.13	13	0.376

### Influence of infection on the baseline immune function indexes

In order to determine and exclude the influence of infection on the baseline immune function indexes, 145 patients with ATB administration were divided into the non-infection group (*n* = 70) and the infection group (*n* = 75). Non-infection patients were treated with antibiotics only for their diagnostic needs, infection patients were prescribed antibiotics because of complications with pulmonary infection. Our results showed that there was no significant difference in the levels of CD3+, CD4+, CD8+, CD4/CD8, CD16 + 56+, CD19, IgM, and D-dimer between the two groups. However, there was a significant difference in L%, NLR, and CRP between the two groups. The results suggested that infection complications in the lung cancer patients enrolled in this study may affect the baseline L%, NLR, and CRP, but had no significant effects on T cell immunity (Table 13). So, this result provided the probability that broad-spectrum antibiotics associated with gut microbiome disturbance, but not infection itself may contribute to impaired T cell immunity.

**Table 13:** Influence of infection on the baseline immune function indexes in lung cancer patients

Characteristic	Non infection group(*n* = 70)	Infection group (*n* = 75)	***P*** value
CD3+ (%)	63.40 (54.99，71.20)	66.20 (55.70，70.90)	0.773
CD4+ (%)	35.76 ± 10.57	33.91 ± 11.34	0.312
CD8+ (%)	20.33 (15.30，24.90)	20.30 (15.27，23.66)	0.818
CD4/CD8	1.78 (1.33，2.34)	1.65 (1.18，2.24)	0.437
CD16 + 56+ (%)	17.40 (11.40，26.14)	18.00 (14.70，24.58)	0.495
CD19+ (%)	9.50 (6.18，14.30)	9.56 (5.76，16.50)	0.880
IgM (g/L)	0.83 (0.56，1.08)	0.84 (0.55，1.13)	0.797
L%	15.70 (9.48，22.78)	11.50 (7.00，18.00)	0.019
NLR	4.74 (2.85，8.81)	6.73 (3.92，11.91)	0.017
CRP (mg/L)	17.11 (6.98，63.95)	58.69 (9.25，111.49)	0.025
D-Dimer (mg/L)	3.01 (0.94，5.95)	1.89 (0.88，4.64)	0.183

*L%* Percentage of lymphocytes, *NLR* Neutrophil-lymphocyte ratio, *CRP* C-reactive protein, *IgM* Immunoglobulin M

## Discussion

Recent studies [8, 11, 14, 31–33] have highlighted the key role of gut microbiota in mediating tumor responses to chemotherapies or immunotherapies. Gui et al. observed that mouse models of lung cancer treated with cisplatin and antibiotics had larger tumors and lower survival rates than those treated with cisplatin alone [25]. In contrast, mice given cisplatin in combination with Lactobacillus responded better to the treatment, which appears to be related to the enhancement of T-cell immunity mediated by commensal microbiota [34]. Overuse of antibiotics may alter the composition of the gut microbiota and have harmful effects on the host. Accumulating evidence has demonstrated that specific microorganisms or microbial disorders promote the progression of hepatic, biliary, and pancreatic tumors by damaging DNA, activating oncogenic signaling pathways, or producing tumor-promoting metabolites [34]. Studies [35, 36] also have shown that the integrity of gut microbiota or Probiotics such as Bifidobacterium is favorable for anti-cancer. Our results have demonstrated that gut microbiota regulates tumor metastasis via non-coding RNA networks [29]. However, whether gut microbiota dysbiosis is involved in the regulation of cancer metastasis in clinical lung cancer patients remains largely unknown.

The purpose of this article was to determine whether gut microbiota dysbiosis due to the administration of ATB impairs T cell immune function and ultimately promotes metastasis in lung patients. Our retrospective analysis showed a significantly shorter MFS in the ATB group compared to the non-ATB group. The influences of ATB were further evaluated according to pathological types such as adenocarcinoma or squamous carcinoma, and these analyses suggest that ATB significantly promotes tumor metastasis in both adenocarcinoma and squamous cell carcinoma of lung cancer.

The 16S rDNA sequencing analysis revealed that Firmicutes abundance is significantly decreased along with increased Proteobacteria and decreased Actinomycetes in the ATB group compared to the non-ATB group. Thus, ATB administration may damage the integrity of gut microbiota including reduction of the probiotics, such as Bifidobacterium and Lactobacillus, which belong to the Actinomycetes or Firmicutes. These changes in turn may promote cancer metastasis.

We found that compared with the non-ATB group, CD3, CD4, CD8, and CD16/56 T cells in the ATB group were significantly decreased. The result of the ROC curve showed CD4, CD8, and CD16/56 have predictive values for MFS, but not D-Dimer, or IgM. These results suggest that long-term broad-spectrum antibiotic administration impairs the clinical benefits in lung cancer patients, either in early staged or advanced lung cancer, and the enhanced metastasis may be attributed to gut microbiome dysbiosis. Therefore, emerging strategies for microbiome control, such as the cautious use of long-term broad-spectrum antibiotics in cancer patients or the consideration of interventions for gut microbiome disorders [37], such as probiotics [38] during chemotherapy or immunotherapy might need to be considered.

In this study, a further stratification of treatment line and typology was performed. Between the non-ATB and ATB groups, our results suggest that there is a significant difference of CD4 T cells in patients who received either first-line therapy or subsequent lines therapy, while CD8 T cells was found to be significant different only in the patients with first-line therapy. Furthermore, CD4 cells but not CD8 T cells were found to be significant different in patients who received either chemotherapy therapy or other treatments. However, due to the limited sample size, for further evaluation and stratification of the treatment line and typology, more studies are needed.

Given that the performance of gut microbiota in cancer has surprised us, it is maybe the prime time to overcome the upcoming challenges in the cancer therapeutic field through more high-quality research. In order to translate the presented results into future clinical possibilities, more samples are needed for subgroup analysis. In addition to lung adenocarcinoma, more pathological types of lung cancer can be analyzed. It is also important to evaluate whether categories of antibiotics or combinations have different effect on cancer. Finally, our study hopefully can raise awareness of careful administration of antibiotics, which is currently a major problem in medicine, not only in associated oncology.

## Conclusions

This study demonstrates a strong interaction between gut microbiota and cancer metastasis, and suggests a potential mechanism linking microbial dysbiosis to cancer progression. Thus, a gut microecological disorder caused by broad-spectrum antibiotics may lead to the imbalance of the human immune system, impair T cell immune function, and cause immune tolerance or immune escape, ultimately promoting cancer metastasis. Therefore, our data suggests the previously unrecognized regulatory potential of the gut microbiome in lung cancer metastasis.

## Data Availability

The 16S rDNA sequencing datasets generated and/or analyzed during the current study are available in the NCBI Sequence Read Archive (SRA) repository (Accession Number: SRP226777).

## Abbreviations

ATB: Broad-spectrum antibiotics
MFS: Metastasis-free survival
NSCLC: Non-small cell lung cancer
ADC: Adenocarcinoma
SCC: Squamous cell carcinoma
NLR: Neutrophil-lymphocyte ratio
CRP: C-reactive protein
ROC: Receiver operating characteristic
AUC: Area under the curve
